# Immunomagnetic‐bead enriched culturomics (IMBEC) for isolating pathobionts from feces of colorectal cancer patients

**DOI:** 10.1002/imt2.100

**Published:** 2023-04-05

**Authors:** Ziran Huang, Yuxiao Chang, Kun Hao, Yafang Tan, Lei Ding, Likun Wang, Zhen Wang, Zhiyuan Pan, Hong Gao, Jiahong Wu, Yubing Zhu, Qi Gao, Yujing Bi, Ruifu Yang

**Affiliations:** ^1^ The Key and Characteristic Laboratory of Modern Pathogen Biology, School of Basic Medical Sciences Guizhou Medical University Guiyang China; ^2^ State Key Laboratory of Pathogen and Biosecurity Beijing Institute of Microbiology and Epidemiology Beijing China; ^3^ Beijing Key Laboratory of POCT for Bioemergency and Clinic (BZ0329) Beijing China; ^4^ Beijing Shijitan Hospital Capital Medical University Beijing China

**Keywords:** colorectal cancer, immunomagnetic‐bead enriched culturomics (IMBEC), magnetic bead‐conjugated antibody, pathobionts, proinflammation

## Abstract

Culturomics employs various cultivating conditions to obtain different types of bacteria and new species. However, current culturomics lacks a highly efficient method for isolating specific pathobionts. Immunomagnetic bead technology, which uses magnetic beads conjugated with antibodies for capturing the antigen to realize enrichment of the targets, has been employed as an alternative method. In this study, we developed a novel method, immunomagnetic bead‐enriched culturomics (IMBEC), in which magnetic bead‐conjugated antibodies purified from the fecal samples of patients with colorectal cancer (CRC) were used to enrich and isolate potential pathobionts. A protocol for enriching potential pathobionts via immunomagnetic capture was developed by optimizing the concentrations of coupling reagents, NaCl, and detergent. The efficacy of pathobiont enrichment was compared between antibody‐coated magnetic beads (antibody group) and nonconjugated blank magnetic beads (blank group). To determine the proinflammatory potential of isolates from both groups, we investigated their ability to induce cytokine production in THP‐1 macrophages. This protocol was employed for isolating bacteria from 10 fecal samples of patients with CRC, which were simultaneously compared with those isolated from the blank group. A total of 209 bacterial species were isolated from both groups, including 173 from the antibody group, 160 from the blank group, and 124 from both groups. Bacteria isolated from the antibody group produced more proinflammatory cytokines than those isolated from the blank group. IMBEC is a promising method for relatively specific isolation of potential pathobionts for a particular disease of interest.

## Highlights

- We have developed a novel method called immunomagnetic bead‐enriched culturomics (IMBEC) to enrich and isolate colorectal cancer‐related pathobionts. This method combines immunomagnetic beads and culturomics technology.
- IMBEC can be targeted to isolate more bacterial species associated with cancer or inflammation, and can be accurate to the strain level.
- Bacterial DNA is not only detected in the blood of colorectal cancer patients but also in the serum of patients with other diseases, so IMBEC can also be employed in other diseases to isolate pathobionts.

## Graphical abstract

We developed a novel method, immunomagnetic bead‐enriched culturomics (IMBEC), which can obtain potential pathobionts from the precultivated colorectal cancer (CRC) fecal samples. We optimized the IMBEC process from the perspective of coupling reagents and magnetic beads washing lotion. Not only in CRC but it has also been reported that antibodies produced by pathobiont strains exist in the blood of patients with other diseases, so the method of precise isolation of disease‐related strains by IMBEC is expected to be a powerful method to accurately isolate pathobiont strains.

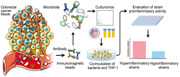

## INTRODUCTION

The Human Microbiome Project has garnered global attention and interest from researchers across various fields, particularly in the study of gut microbes in the biomedical field. Studies have demonstrated the association of gut microbiota with chronic diseases such as gastrointestinal inflammation, metabolic diseases, cardiovascular diseases, and cancer [1, 2, 3, 4]. However, most research has relied on sequencing techniques to identify potential pathobionts and decipher the mechanisms of bacteria–host interactions [5, 6]. Dr. Hajishengallis proposed that normally harmless symbionts could become pathogenic under certain environmental conditions, such as immune system disorders or disruption of homeostasis [7]. Current microbiome research aims to elucidate the role and mechanisms of the microbiome in host health and disease [2, 8, 9, 10], which is a significant inflection point in the research of the human microbiome. Although potential pathobionts are identified through sequencing correlation analysis, the target bacteria are obtained from culture collection centers for further investigation. Ideally, potential pathobionts should be isolated directly from study participants, but current culturomics methods are inefficient for obtaining specific pathobionts. Thus, novel methods for isolating specific pathobionts are necessary.

The intestines have mechanical, biological, chemical, and immune barriers, and only a well‐functioning intestinal barrier can ensure the normal functioning of most organs and systems in the body [11]. Studies on the mechanisms of gut microbes and diseases have revealed that the common pathogenic mechanism of the gut microbiome is the induction of chronic inflammation [12, 13, 14, 15]. This involves dysbiosis of the intestinal microbiome and disruption of gut barriers, leading to the enrichment of certain pathobionts that produce virulent factors and unfavorable metabolites, eliciting immune responses and low‐grade chronic inflammation. Over time, chronic inflammation can exacerbate gut dysbiosis and instigate a malignant cycle [16]. In this study, we aimed to determine whether pathobiont‐induced antibodies in patients could be used to label magnetic beads for developing a pathobiont‐enriched culturomics technique for the efficient isolation of target bacteria.

Immunomagnetic bead technology conjugates active proteins, such as antibodies, to the surface of chemically modified magnetic beads to form immunomagnetic beads. These beads bind to target substances, such as antigens, to form an antigen–antibody immunomagnetic bead complex, achieving directional movement through the action of magnetic fields, and thus achieving enrichment [17, 18]. Therefore, we developed a novel method called immunomagnetic bead‐enriched culturomics (IMBEC) to enrich and isolate colorectal cancer (CRC)‐related pathobionts. The gut microbiome is involved in the development of CRC [15, 19, 20]. Moreover, higher levels of antibodies against *Fusobacterium nucleatum* have been detected in the serum of patients with CRC than in healthy controls [21]. This finding suggests that bacteria or their metabolites in patients with CRC may interact with immune cells and induce antibody production. To develop the IMBEC method, we purified antibodies from patients with CRC and conjugated them with magnetic beads for the enrichment and isolation of potential pathobionts from precultured fecal samples. Moreover, we evaluated the proinflammatory potentials of the isolates by detecting cytokine production in THP‐1 cells.

## RESULTS

### Optimization of antibody‐coupled immunomagnetic beads and culturomics procedure

To achieve optimal antibody‐coated beads, we optimized the concentrations of coupling reagents, including *N*‐hydroxysuccinimide (NHS) and 1‐ethyl‐3‐(3‐dimethylaminopropyl) carbodiimide (EDC), and antibodies for bead preparation. We assessed the results by determining the concentration of coupled antibodies. Our findings revealed that a higher concentration of EDC (52.2 mM) was more effective than a lower concentration (26.1 mM). In contrast, higher concentrations of NHS decreased the coupling effect. Therefore, the 0 mM NHS + 52.2 mM EDC treatment group exhibited the best coupling effect and could couple 136 μg of antibody (Table 1). We also identified the saturation concentration of antibody required for coupling, which was determined to be 0.160 mg of antibody coupled with 0.138 mg of the antibody to 5 mg of magnetic beads (Table 2), chosen as the final antibody coating protocol.

**Table 1:** Optimization effect of coupling reagent concentration.

Group (NHS + EDC mM)	BCA concentration of uncoupled antibody (mg/mL)	Amount of uncoupled antibody (mg)	Amount of coupled antibody (mg)
0 + 26.1	0.369	0.207	0.114
0 + 52.2	0.329	0.184	0.136
86.9 + 26.1	0.425	0.238	0.082
86.9 + 52.2	0.376	0.211	0.109
174 + 26.1	0.462	0.259	0.062
174 + 52.2	0.459	0.257	0.064

Abbreviations: BCA, bicinchoninic acid assay; EDC, 1‐(3‐dimethylaminopropyl)‐3‐ethylcarbodiimide hydrochloride); NHS, *N*‐hydroxysuccinimide.

**Table 2:** Optimal antibody coupling volume.

Group (μL of antibody)	BCA concentration of uncoupled antibody (mg/mL)	Amount of uncoupled antibody (mg)	Amount of coupled antibody (mg)
15	0.007	0.003	0.077
20	0.010	0.005	0.102
25	0.024	0.013	0.121
30	0.042	0.022	0.138
35	0.091	0.049	0.138
40	0.142	0.076	0.138

Abbreviation: BCA, bicinchoninic acid assay.

**Figure 1:**
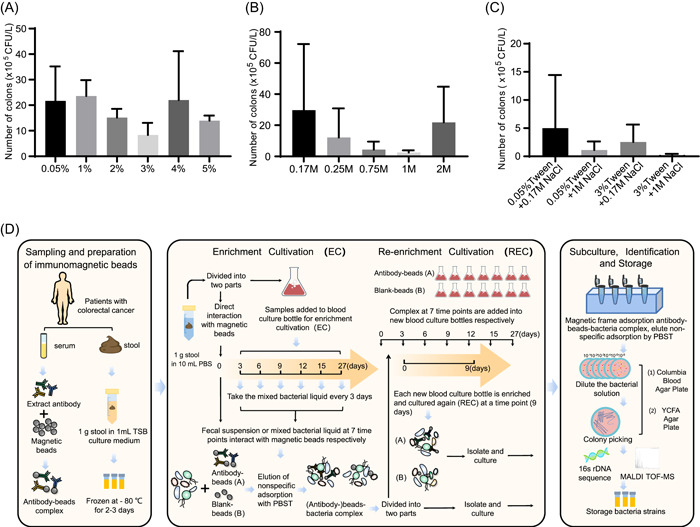
Procedure of immunomagnetic bead‐enriched culturomics and its optimization. (A–C) Concentration of Tween and NaCl in phosphate‐buffered saline with Tween‐20 (PBST) buffer was optimized to reduce the nonspecific binding of magnetic beads. (A) Eluting effect of different concentrations of Tween in PBST on nonspecific adsorption. (B) Eluting effect of different concentrations of NaCl in PBST on nonspecific adsorption. (C) Screening of the optimal combination of Tween and NaCl in terms of the eluting effect. (D) Entire process of immunomagnetic‐bead enriched culturomics (IMBEC): stool and blood samples were collected from patients with colorectal cancer (CRC), and antibodies from the corresponding serum were extracted and coated onto the surface of magnetic beads to form antibody beads; stool samples were resuspended in TSB medium and briefly frozen at −80°C if not cultured on time. Subsequently, 1 g of fresh stool sample was resuspended in 10 ml of PBS. The fecal suspension was divided into two parts: one part was incubated with antibody beads (antibody group, A) or blank beads (blank group, B) on Day 0, while the other part was precultured in an anaerobic blood bottle containing 5% defatted sheep blood and 5% rumen fluid for 27 days (enrichment culture, EC) at a certain interval (Days 3, 6, 9, 12, 15, and 27). The bacteria in the EC were incubated with antibody beads or blank beads to form bacteria–antibody–magnetic beads complex. The complex was divided into two parts, one of which was directly diluted and spread onto blood agar or yeast casitone fatty acid (YCFA) plates and incubated under anaerobic conditions at 37°C for 3 days. The monoclonal colonies were identified via matrix‐assisted laser desorption/ionization time‐of‐flight mass spectrometry (MALDI‐TOF MS) and 16S ribosomal DNA (rDNA) sequencing, and the strains were stored at −80°C. The other part of the complex was stored in new anaerobic blood bottles for 9 days again (re‐enrichment culture, REC), and the subculture suspension was isolated and identified as previously described. CFU, colony‐forming unit.

To minimize the nonspecific binding of magnetic beads, we optimized the concentration of Tween‐20 and NaCl in the phosphate‐buffered saline (PBS) with Tween‐20 (PBST) washing solution. We found that using 3% Tween‐20 and 1 M NaCl was the most optimal concentration to minimize nonspecific binding (Figure 1A,B). Additionally, we tested multiple concentrations of Tween‐20 and NaCl and found that the combination of 3% Tween‐20 and 1 M NaCl produced the best elution results (Figure 1C).

The immunomagnetic bead enrichment culturomics (IMBEC) procedure, shown in Figure 1D, involved collecting fecal samples, which were divided into two parts. One part was reacted with antibody‐coated beads (group A) or blank beads (group B) on Day 0, while the other part was precultured in anaerobic blood bottles for 27 days (EC). On Days 3, 6, 9, 12, 15, and 27, the preculture was collected and mixed with antibody or blank beads for bacterial enrichment to form an antibody–bead–bacteria complex. After elution, one part of the complex was directly diluted and spread onto blood agar or yeast casitone fatty acid (YCFA) plates, and individual colonies were identified via matrix‐assisted laser desorption/ionization time‐of‐flight mass spectrometry (MALDI‐TOF MS) or 16S ribosomal DNA (rDNA) sequencing [22]. The other part of the complex was stored in new anaerobic blood bottles for re‐enrichment cultivation (REC) to increase the number of low‐abundance bacteria. After 9 days of REC, the bacteria in the suspension were isolated and identified as previously described. Finally, all colonies were stored at −80°C for further use.

### Isolation of bacteria from fecal samples of patients with CRC

**Figure 2:**
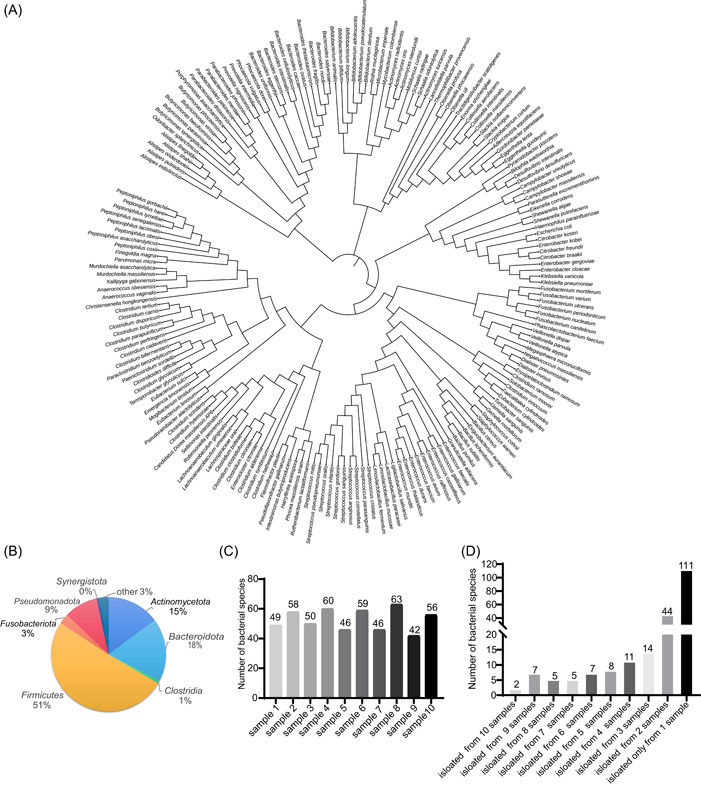
Composition of strains isolated from 10 fecal samples of colorectal cancer (CRC) patients. (A) 16S ribosomal DNA (rDNA)‐sequencing‐based evolutionary tree of 209 bacterial species isolated from the antibody and blank groups. (B) Phylum‐level bacterial composition of 209 bacterial species. (C) Total number of species isolated from each sample. (D) Numbers of bacterial species isolated from different numbers of sample(s). A, antibody group; B, blank group.

We used IMBEC to isolate bacteria from 10 fecal samples from patients with CRC. A total of 209 bacterial species were isolated from 10 samples (Supporting Information: Figure S1 and Figure 2A), which included six novel species (Supporting Information: Table S1). The most prevalent bacterial phyla were *Firmicutes, Bacteroidota, Actinomycetota*, and *Pseudomonadota*, accounting for most of the isolated bacteria (Figure 2B). At the genus level, the bacterial composition between the antibody and control groups was generally consistent, with some exceptions. Notably, the antibody group had a higher proportion of *Adlercreutzia, Atopobium, Campylobacter, Rothia, Alistipes, Christensenella, Dialister, Citrobacte*r, *Eikenella, Granulicatella, Shewanella*, and *Sellimonas* (Supporting Information: Figure S2A). We isolated approximately 50–60 bacterial species from each sample (Figure 2C), with nearly half of the species being unique to a single sample, indicating a high degree of sample diversity (Figure 2D). Of the 209 bacterial species isolated, 49 were exclusively found in the antibody group, 36 in the control group, and 124 in both groups (Supporting Information: Figure S2B).

### Immunomagnetic bead‐enriched preculture increases bacteria isolation yield

**Figure 3:**
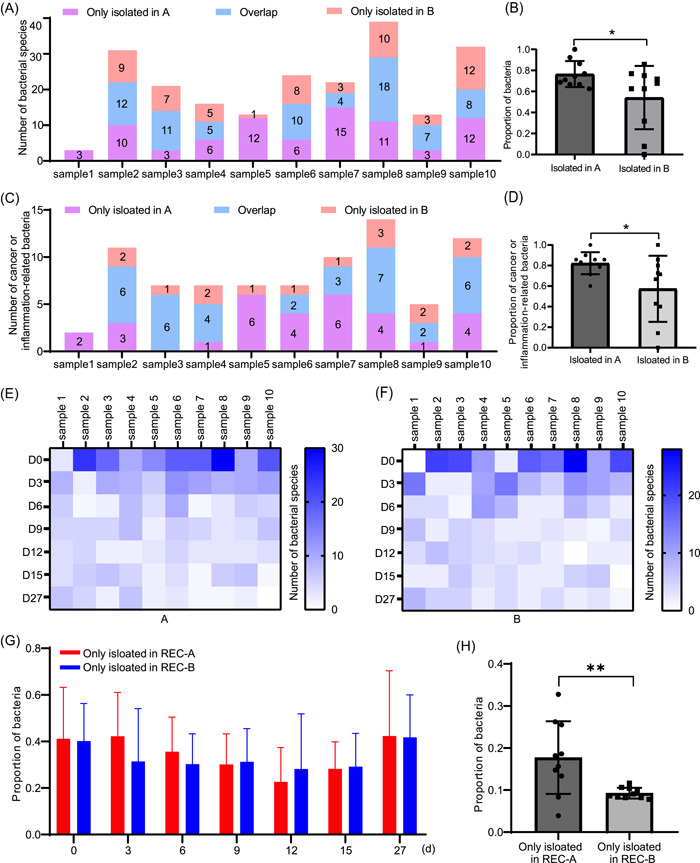
Comparison of bacteria isolated under different culture conditions with or without increasing the preculture time. (A)–(D) Bacteria isolated at Day 0, that is, directly isolated from fecal samples. (A) Number of bacteria isolated per sample from A or B. (B) Proportion of bacteria isolated from A or B. (C) Number of cancer‐ or inflammation‐related bacteria isolated from each sample from A or B. (D) Proportion of cancer‐ or inflammation‐related bacteria isolated from A or B. Data are presented as the mean ± SD in (B) and (D) and were analyzed using two‐tailed unpaired Student's *t*‐test, *p < 0.05. (E)–(H) Bacterial strains isolated for each sample with prolonged preculture time. (E) Heat map of new species isolated from A at seven time points. (F) Heat map of new species isolated from B at seven time points. (G) Proportion of bacteria isolated at seven time points from REC‐A or REC‐B. (H) Proportion of bacteria isolated only from REC‐A or REC‐B to the total number of bacteria in each sample under REC. Data are presented as the mean ± SD in (G) and (H) and were analyzed using two‐tailed unpaired Student's *t*‐test, *p < 0.05; **p < 0.01. A, antibody group; B, blank group; REC, re‐enrichment cultivation; REC‐A, re‐enrichment cultivation of antibody group; REC‐B, re‐enrichment cultivation of blank group.

We compared the bacteria isolation results from fecal samples under different experimental conditions. On Day 0, the antibody group had 27 isolated species, the blank group had 18, and 35 were isolated from both groups under the enrichment cultivation (EC) condition (Supporting Information: Figure S3A). Under both EC and REC conditions, 30 species were isolated from the antibody group, 29 from the blank group, and 56 from both groups (Supporting Information: Figure S3B). Statistical analysis revealed that the bacterial species isolated from the antibody and blank groups on Day 0 were different (Figure 3A), with a significantly higher number of bacterial species isolated from the antibody group (Figure 3B).

We then compared the number of bacteria associated with cancer or inflammation in both groups. Using PubMed, we searched for the 209 species of bacteria isolated from the 10 samples and identified 47 species related to cancer or inflammation based on existing experimental or bioinformatics data (Supporting Information: Table S2). On Day 0, we were able to isolate a significantly higher number of pathobionts from the antibody group compared to the blank group (Figure 3C,D). Increasing the preculture time allowed us to identify and isolate additional bacterial strains, regardless of the group (Figure 3E,F). At each time point, we could isolate previously unobserved strains. Therefore, it is necessary to extend the incubation time.

Furthermore, more than 30% of the strains were isolated only under the EC condition in both the antibody (EC‐A) and blank (EC‐B) groups (Supporting Information: Figure S3C). REC was utilized to culture low‐abundance bacteria, and we were able to isolate additional bacterial strains at each time point (Figure 3G), with over 20% of the strains being exclusively isolated under the REC condition in both groups (Supporting Information: Figure S3D). Therefore, EC and REC were found to be complementary and indispensable, with REC isolating significantly more bacterial species from the antibody group (REC‐A) than from the blank group (REC‐B; Figure 3H).

### Immunomagnetic bead capture significantly enhanced the isolation efficiency of proinflammatory or cancer‐promoting bacteria

**Figure 4:**
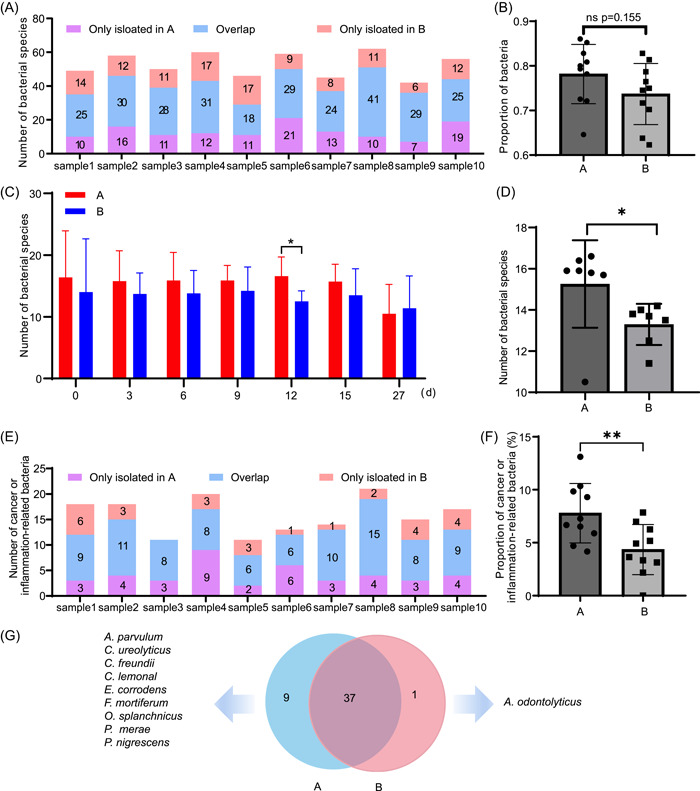
Comparison of the number of bacterial species or cancer‐ or inflammation‐related bacterial species isolated from the antibody and blank groups. (A)–(D) Number of bacterial species isolated from A or B. (A) Number of species isolated from each sample from A or B. (B) Proportion of species isolated from A or B to the total number of bacteria in the 10 samples. (C) Number of species isolated from A or B at seven time points. (D) Statistical analysis of the number of species isolated from A or B at seven time points. (E and F) Number of bacterial species associated with cancer or inflammation isolated from A or B. (E) Number of cancer‐ or inflammation‐related bacterial species isolated from each sample from A or B. (F) Statistical analysis of the percentage of cancer‐ or inflammation‐related bacteria isolated from A or B of each sample. (G) Distribution of 47 cancer‐ or inflammation‐related species isolated from A and B. Data are presented as the mean ±SD and were analyzed using two‐tailed unpaired Student's *t*‐test in (B)–(D) and (F), *p < 0.05; **p < 0.01; ns, no significance. A, antibody group; B, blank group.

To assess differences in bacterial isolation following antibody magnetic bead capture, we analyzed all bacteria at all time points. Blank magnetic beads, without antibody coating, were used as controls to exclude nonspecific binding. As indicated by the results of each sample, some bacteria were also isolated from the blank group (Figure 4A). While more bacterial species were isolated from the antibody group than from the blank group, the difference was not significant (Figure 4B). However, according to the time point statistics, a significantly higher number of bacterial species were isolated from the antibody group than from the blank group at each time point except for the last (Figure 4C,D and Supporting Information: Figure S4).

Next, we compared the number of bacteria associated with cancer or inflammation in both groups. In each sample, significantly more cancer‐ or inflammation‐related bacteria were isolated from the antibody group (Figure 4E) than from the blank group (Figure 4F). Among the 47 bacterial species isolated, 9 were isolated from the antibody group alone, 1 from the blank group alone, and 37 from both groups (Figure 4G). These findings suggest that while the magnetic beads exhibited nonspecific binding, the antibody group demonstrated efficient isolation of disease‐related pathobionts.

### Bacteria isolated from the antibody group demonstrated higher proinflammatory potential

To confirm that the bacteria isolated from the antibody group were related to CRC, some of the strains associated with cancer or inflammation were incubated with THP‐1 cells to assess their potential to induce interleukin‐1β (IL‐1β), IL‐6, and tumor necrosis factor (TNF‐α) production. The following criteria were used to select experimental strains from all isolates (Supporting Information: Figure S5A,B): (1) bacterial species previously reported to be associated with cancer or proinflammation and (2) equal proportion of experimental strains in the two groups. As a result, 24 species and 106 strains (listed in Supporting Information: Table S3) were selected for further evaluation of cytokine induction in THP‐1 cells.

**Figure 5:**
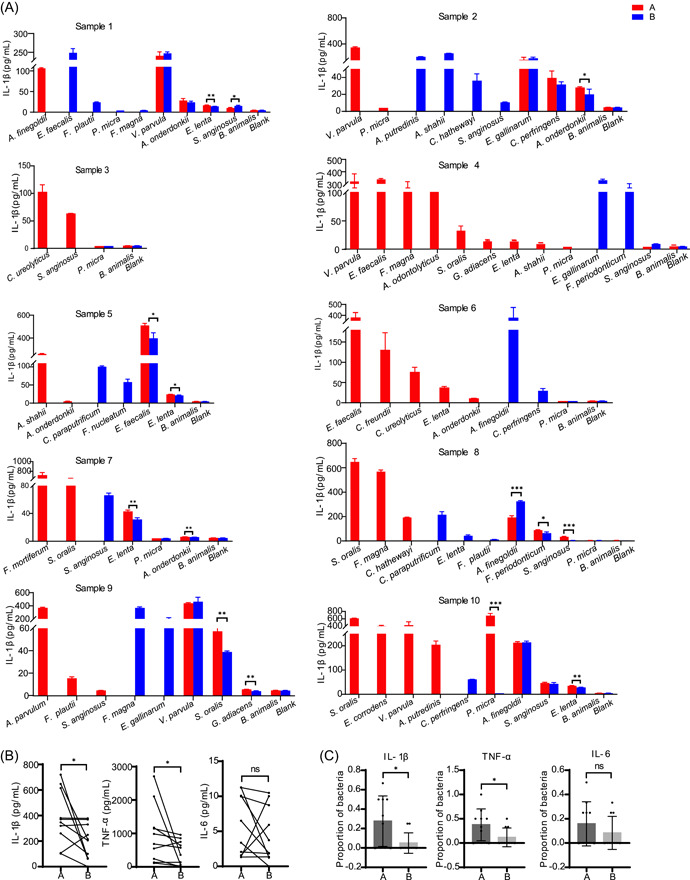
Comparison of the proinflammatory ability of cancer‐ or inflammation‐related strains isolated from antibody and blank groups. (A) Interleukin‐1β (IL‐1β) concentration in the supernatant of 106 strains coincubated with THP‐1. One column alone means only isolated in the antibody group or blank group; two columns mean isolated in both groups. Data are presented as the mean ± SD and were analyzed using two‐tailed unpaired Student's *t*‐test, *p < 0.05, **p < 0.01, and ***p < 0.001. (B) Comparison of the strains with the strongest inflammatory ability isolated from A or B alone; these strains were selected from each sample, and the levels of cytokines stimulated by these strains were compared between A and B. Data analysis was performed using two‐tailed paired Student's *t*‐test, *p < 0.05; ns, no significance. (C) Comparison of the strain‐level proinflammatory ability of isolates from shared parts between A and B. Data are presented as the mean ± SD and were analyzed using two‐tailed unpaired Student's *t*‐test, *p < 0.05; ns, no significance. A, antibody group; B, blank group; IL, interleukin; TNF‐α, tumor necrosis factor‐α.

All experimental strains were incubated with THP‐1 cells to detect TNF‐α, IL‐1β, and IL‐6 concentrations in the cell supernatant (Figure 5A and Supporting Information: Figures S6 and S7). First, we compared the proinflammatory abilities of the experimental strains only isolated in the antibody (A) and blank (B) groups. The strains with the strongest proinflammatory ability isolated from the A or B group alone were selected from each sample. Through the paired comparison of 10 samples between both groups, we found significantly higher levels of TNF‐α and IL‐1β in the antibody group than in the blank group. However, no significant difference in IL‐6 level was observed between the two groups (Figure 5B).

Next, we analyzed and compared the intensity of inflammation induced by different strains of the same species isolated in both groups. The results revealed inconsistency in cytokine induction between the two groups. The strains isolated from the antibody group produced higher levels of IL‐1β and TNF‐α in THP‐1 cells than strains isolated from the blank group (Figure 5C); however, this was not the case with IL‐6. The antibody group exhibited higher inflammatory potential regardless of the comparison of isolates found only in the antibody or blank group or of shared strains. Taken together, our findings suggest that IMBEC could identify more proinflammatory pathobionts strains from the fecal samples of patients with CRC.

## DISCUSSION

The intestinal microbiota plays a crucial role in the occurrence and development of CRC, with various bacteria being identified as potential pathobionts of CRC [23, 24, 25]. The interaction between the microbiota and the host is highly complex. To gain a deeper understanding of the role of bacteria in CRC, it is imperative to isolate pathogenic bacteria associated with CRC. However, efficiently obtaining CRC‐related pathobionts remains a challenging task. With the rapid advancements in sequencing technology, 16S rDNA sequencing and metagenomics have become widely used to elucidate the association between pathobionts and diseases [26, 27, 28]. Nonetheless, existing results appear inconsistent across different patient groups due to differences in sequencing platforms and analytical tools, individual differences, and the complexity of intestinal microorganisms [10]. Several limitations of sequencing technology can be overcome using culturomics, which employs various culture conditions to identify bacteria through MALDI‐TOF MS and 16S rDNA sequencing.

Culturomics can be used to isolate bacterial strains for further experimental validation [29]. Previous studies have shown that different strains of the same species may exhibit different virulence and functional potential [30], making it essential to isolate pathobiont strains for studying the relationship between bacteria and disease. However, conventional culturing methods may not accurately identify the target bacteria. Researchers have developed reverse genomics‐based methods for bacterial isolation that employ antibodies targeting specific epitopes of bacterial membrane proteins to isolate specific bacterial species from the microbiota. This technique enabled the successful isolation of the human oral bacterium SR1, which could not be cultivated previously [31]. Additionally, Raman‐activated cell sorting sequencing technology has emerged as a targeted screening tool for the metabolic function of bacteria and screen functional strains, which uses functional sorting of cells based on a single‐cell Raman spectrum [32]. However, these techniques may not be suitable for relatively specific targeted isolation of pathogenic bacteria. Immunomagnetic bead‐based techniques have long been used for sample purification and enrichment, with their primary application being the rapid detection of food pathogens [33]. In IMBEC technology, patient‐derived antibodies are coated with magnetic beads to form immunomagnetic beads, which are then subjected to culturomics for the isolation and cultivation of pathobionts. IMBEC is a promising technique for the targeted isolation of pathogenic bacteria.

**Figure 6:**
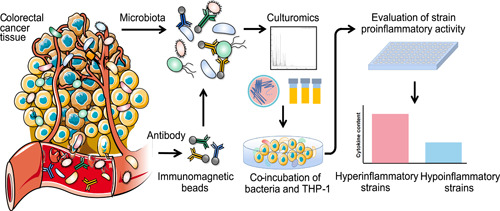
Schematic diagram of the immunomagnetic‐bead enriched culturomics (IMBEC) technology. Colorectal cancer (CRC) tumor tissue invades the intestinal wall and induces inflammation, which increases the permeability of the intestinal wall, resulting in the translocation of pathobionts and their metabolites to trigger immune responses of the host, which in turn stimulates the body's immune system to produce corresponding antibodies. Antibodies in the blood of a CRC patient are extracted and coated on the surface of magnetic beads to form immunomagnetic beads, which are incubated with the patient's stool sample such that CRC‐related pathobionts can be accurately screened, isolated, and identified by culturomics.

In this study, we employed a novel method utilizing magnetic bead materials and the principle of antigen–antibody binding to develop the IMBEC approach for identifying pathobionts. Patients with CRC exhibit increased intestinal permeability, which leads to dysbiosis of the gut microbiota and tumor growth, disrupting the integrity of the intestinal wall and potentially displacing bacteria or metabolites in tumor tissues and microenvironments [34, 35]. This disruption elicits an immune response involving antibody production to resist bacterial invasion and maintain body homeostasis. Populations of some intestinal bacteria shift with lymphocytes, macrophages, and Paneth cells [36]. Furthermore, other studies have identified more bacterial DNA in the peripheral blood of CRC patients compared to healthy controls [37], indicating a potential relationship between these bacteria and CRC. As such, we hypothesized that antibodies produced in patients could be used to coat magnetic beads for capturing CRC‐related pathobionts. To develop the IMBEC approach for efficient isolation of pathobionts from fecal samples of patients with CRC, we utilized immunomagnetic bead technology and culturomics. The magnetic beads were coated with patients’ own antibodies and then bound to pathobionts in precultivated fecal samples in anaerobic blood bottles to form a bacteria–antibody–magnetic bead complex. This complex was then cultivated via culturomics to efficiently isolate CRC‐associated pathobiont strains (Figure 6).

Results showed significantly more bacteria and a higher proportion of bacterial species were isolated from the antibody group than from the blank group. Fine particles in the fecal suspension can result in nonspecific binding of the magnetic beads; however, antibodies stimulated by bacteria possess a certain affinity to the bacteria itself [37]. Therefore, the immunomagnetic beads can still bind specifically to pathobiont bacteria. The antibody group also produced significantly more cancer‐ or inflammation‐related bacterial species than the blank group, indicating that IMBEC can be used to identify more disease‐associated pathobionts. Regardless of whether the tumor is caused by inflammation or inflammation is caused by the tumor, inflammatory substances infiltrate the tumor tissue and promote further tumor development [12]. The bacteria isolated from the antibody, or the blank group alone, were incubated with THP‐1 cells. Regarding virulence, few strains isolated from the blank group were more virulent than those isolated from the antibody group. The reason may be that the antibody coating was not type‐specific, including IgG, IgA, and so on. IgA may be more stimulated by antigens expressed by symbiotic bacteria [38]. However, most bacteria isolated from the antibody group were more virulent than those isolated from the blank group. A direct comparison cannot truly reflect their virulence because bacteria isolated only from the antibody or blank group belong to different species and have different virulence. Therefore, we compared only the most virulent strains isolated only from the antibody group or the blank group, and bacteria isolated only from the former demonstrated stronger proinflammatory effects than those isolated only from the latter. Additionally, we analyzed and compared the intensity of inflammation induced by strains shared by the two groups, highlighting strain‐level differences and the need to examine disease–pathobiont relationships at the strain level. In conclusion, the proinflammatory effect of bacterial strains isolated using immunomagnetic beads was strong, indicating that IMBEC can serve as a general method for identifying pathobionts for any disease of interest.

In recent years, bacterial DNA has been detected not only in the blood of patients with CRC but also in the serum of patients with other diseases. For instance, quantitative analysis of bacterial DNA isolated from the blood samples of patients with metabolic diseases has revealed that blood bacterial characteristics are related to metabolic health. Scores based on the phyla and genera of bacteria detected in the blood could accurately distinguish patients with metabolic syndrome from those with non‐metabolic syndromes [39]. Similarly, sequencing analysis of blood samples from patients with cardiovascular disease has shown significantly higher levels of bacterial DNA in these patients than in controls, with varying bacterial composition [40]. Furthermore, a recent study identified 65 bacterial species in the portal venous, central venous, and peripheral blood obtained from patients with decompensated cirrhosis [41]. Therefore, IMBEC is expected to serve as a powerful method for the precise isolation of pathobiont strains in many diseases.

However, this study has some limitations. Magnetic beads exhibit strong nonspecific adsorption ability, which can affect the accuracy of pathobiont isolation. In future studies, optimization of the washing solution, use of magnetic beads with different chemically modified groups, magnetic bead diameter size, and other experimental parameters may help reduce the nonspecific binding of magnetic beads and enhance the accuracy of disease‐related pathobiont isolation.

## CONCLUSION

The protocol of IMBEC has been optimized, and the efficacy of pathobiont isolation was found to be higher in the antibody group than in the blank group. Furthermore, strains isolated from the antibody group showed a higher proinflammatory potential than those from the blank group. These results indicate that IMBEC is a promising method for isolating potential pathobionts of many diseases, including CRC.

## METHODS

### Samples and cells

#### Stool and serum samples

Ten patients with CRC at Beijing Shijitan Hospital were included in this study, and stool and blood samples were collected from them. The following criteria were used for patient inclusion: (1) CRC confirmed by endoscopy and histology; (2) no treatment with antibiotics or other drugs that affect the gut microbiome; (3) an indication for surgery; and (4) no consumption of a probiotic‐containing diet. All patient information has been provided in Supporting Information: Table S4. Fresh preoperative stool samples were collected in sterile centrifuge tubes and transferred to the laboratory for processing under anaerobic conditions. Additionally, blood samples were collected following a standardized protocol, and antibodies were stored at −80°C. For culturomics, 1 g of feces was resuspended in 10 mL of tryptone soya broth (3320287; Oxoid). Moreover, 5 mL of blood was collected from each patient for antibody extraction. This study was approved by the Ethics Committee of Shijitan Hospital (Beijing, China; agreement no. 21KY105).

#### Cell line

THP‐1 cells were purchased from Beina Biological Co., Ltd. and cultured in RPMI‐1640 medium (8122151; Gibco) containing 10% fetal bovine serum (8122151; Gemini Bio Product) at 37°C under a 5% CO_2_ atmosphere.

### Preparation of immunomagnetic beads

First, Beijing Hotgen Biotechnology Co., Ltd. conducted morphological monitoring of magnetic bead particles. Transmission electron microscopy showed good homogeneity of the magnetic beads (Supporting Information: Figure S8). Antibodies were extracted from the serum of patients with CRC using octyl‐ammonium sulfate. Briefly, 3‐μm‐diameter hydroxylated magnetic beads (MS300/carboxyl; MBL Beijing Biotech Co., Ltd.) were mixed with the antibodies by shaking well. Next, 5 mg of beads were placed in an Eppendorf tube. The supernatant was removed after magnetic separation, and the beads were washed once with a binding buffer and then mixed ultrasonically for 30 s. Subsequently, a coupling reagent (NHS and EDC) was added, and the solution was mixed at room temperature for 30 min by shaking well. The supernatant was removed after magnetic separation. Thereafter, a binding buffer was added to the supernatant and mixed by shaking well, following which antibodies extracted from the patients with CRC were added. The solution was mixed ultrasonically. After magnetically separating the beads, the supernatant was removed, a cleaning buffer was added, and the beads were washed four times. Finally, 5 mL of a storage solution was added, and the beads were mixed at room temperature for 2 h and stored in a refrigerator at 4°C to obtain immunomagnetic beads (antibody beads).

### Optimization of coupling reagents and magnetic bead washing solution

To optimize the concentrations of coupling reagents, that is, NHS and EDC, we tested two EDC concentrations of 26.1 and 52.2 mM and three NHS concentrations of 0, 86.9, and 174 mM. Purified serum antibody (5.34 mg/mL) was coupled with 5 mg of magnetic beads using approximately 60 μL of the antibody. To determine the optimal amounts of magnetic beads to be coupled with the antibodies, we used 15, 20, 25, 30, 35, and 40 μL of purified serum antibody samples with 5 mg of magnetic beads. The unconjugated antibody was used as a control to determine the optimal amount of the conjugated antibody to be used in the final protocol.

To reduce the nonspecific binding of magnetic beads, blank magnetic beads were incubated with healthy human feces. The bacteria–magnetic bead complex was washed five times with PBST, using different concentrations of NaCl (0.25, 0.75, 1, and 2 M) or different concentrations of Tween (1%, 2%, 3%, 4%, and 5%). PBST containing 0.05% Tween was used as the control. To isolate bacteria, double‐diluted bacteria–magnetic bead complexes were spread onto blood agar plates (PO0123A; Thermo Fisher Scientific) and incubated at 37°C under anaerobic conditions for 3 days. The number of colonies on the plates was counted to evaluate the effects of different washing solutions.

### Cultivation, isolation, and identification of bacteria

#### Bacterial cultivation and isolation

Fecal suspensions were precultured in anaerobic blood bottles containing 5% defatted sheep blood (H0011; Beijing Bote Medical Co., Ltd.) and 5% rumen fluid (Elite Media) for 27 days under anaerobic conditions at 37°C. EC was performed by incubating the preculture mixture with antibody beads (group A) or blank beads (group B) at specific intervals (direct fresh feces were used on Day 0, and the preculture mixture was used on Days 3, 6, 9, 12, 15, and 27) to facilitate the formation of the bacteria–antibody magnetic bead complex. Subsequently, a part of the complex was double diluted and spread onto blood agar or YCFA plates (blood agar plates enable the widespread growth of a large number of bacterial species, while YCFA plates enable the culture of several bacteria found in the human gut microbiota) [42, 43, 44] for anaerobic cultivation at 37°C for 72 h [45]. Colonies were selected based on their characteristics (size, color, and morphology) for subculturing in a modified brain heart infusion broth liquid medium (BHI, 299070; Becton Dickinson and Company) containing 5% sheep blood or YCFA liquid medium. Another part of the complex was placed in new anaerobic blood culture bottles for recultivation for 9 days, known as REC, and the bacterial isolation procedure was repeated.

#### Incubation of immunomagnetic beads with feces

The antibody or blank beads were mixed with fecal suspension (excluding large fecal dregs) or the precultivated bacterial suspension in a ratio of 400:1000 μL. The mixture was incubated at 37°C for 40 min, with intermittent mixing every 8 min to prevent magnetic bead precipitation. The bacteria–antibody magnetic bead complex was separated from unbound bacteria using magnetic field forces and washed five times with an optimized PBST solution (3% Tween and 1 M NaCl).

### Bacterial identification

MALDI‐TOF MS was utilized to identify the bacteria using an Autof MS1000 system (Autobio). Monoclonal colonies were coated on a special plate for mass spectrometry, and lysing buffer (70% formic acid) was added to lyse the bacteria. After natural drying, the matrix buffer (50% acetonitrile and 2.5% trichloroacetic acid) was added. If the score was ≥9.0, the result was considered credible. Samples with a score of <9.0 underwent 16S rDNA sequencing for identification.

For bacterial identification using 16S rDNA sequencing, bacterial DNA was extracted using a QIAamp DNA Mini kit (250) (51306). Polymerase chain reaction and sequencing were performed using primers 27F: 5′‐AGAGTTTGATCCTGGCTAG‐3′ and 1492R: 5′‐GGTTACCTTGTTACGTT‐3′. If the 16S rDNA sequence exhibited <98.65% similarity to that of a known species, it would be considered a potentially novel species [46].

### Cell infection

THP‐1 cells were seeded in 12‐well plates at a density of 5 × 10^5^ cells per well and incubated with 100 ng/mL of phorbol 12‐myristate 13‐acetate (Thermo Fisher Scientific) at 37°C for 48 h in a humidified environment containing 5% CO_2_ to induce their differentiation into macrophages. Bacterial strains associated with inflammation or cancer, *F. nucleatum* (positive control) and *Bifidobacterium animalis* (negative control), were cultivated anaerobically at 37°C until they reached the end of the logarithmic growth phase. Samples of each strain were centrifuged to remove the bacterial medium and then resuspended in a cell culture medium. Subsequently, the bacterial strains were added to the 12‐well plates at a multiplicity of infection of 10 for 8 h, with the first 2 h in an anaerobic incubator and the last 6 h in an incubator containing 5% CO_2_. After infection, the supernatants were collected, centrifuged, and stored at −80°C.

### Enzyme‐Linked immunosorbent assay (ELISA)

The concentrations of IL‐1β, TNF‐α, and IL‐6 produced by bacteria‐stimulated THP‐1 cells were quantified using a precoated ELISA kit (Dakewei; human IL‐1β, 1110122; human TNF‐α, 1117202; and human IL‐6, 1110602) according to the manufacturer's instructions. Each sample was added to three wells, and 100 μL of the sample and 50 μL of biotinylated antibody were added to each well and incubated at room temperature for 3 h. After washing with the lotion three times, 100 μL of streptavidin–horseradish peroxidase antibody was added to the reaction well, and the sample was further incubated at room temperature for 20 min. After washing three times, 3,3′,5,5′‐tetramethylbenzidine solution was added and incubated at room temperature for 15 min before the addition of the stop solution. The optical density of all samples was measured at 450 nm (reference wavelength 620–630 nm) using Molecular Devices SpectraMax (M2). The concentrations of IL‐1β, TNF‐α, and IL‐6 were calculated from the standard curve for each sample.

### Statistical methods

All statistical analyses were performed using GraphPad Prism 8.0 (GraphPad Software, Inc.). Student's *t*‐test and paired *t*‐test were used to compare the variables of the two sample groups. One‐way analysis of variance was used to analyze the data of more than three groups. Data are expressed as the mean ± SD from three independent experiments. All statistical tests were two‐sided, and a *p* value of less than 0.05 was considered statistically significant (*p < 0.05, **p < 0.01, ***p < 0.001).

## AUTHOR CONTRIBUTIONS

Ruifu Yang conceived the study. Ruifu Yang, Yujing Bi, Lei Ding, Jujia Wu, and Qi Gao designed the experiments. Ziran Hang and Yuxiao Chang carried out the experiments and analyzed the data. Kun Hao and Qi Gao performed the antibody purification and magnetic beads coating. Zhen Wang and Likun Wang carried out the experiments. Yafang Tan and Zhiyuan Pan investigated the literature. Biyun Zhu, Lei Ding, and Hong Gao collected the samples. Ziran Hang, Yuxiao Chang, and Yujing Bi wrote the manuscript. Ruifu Yang revised the manuscript.

## CONFLICT OF INTEREST STATEMENT

The authors have declared no competing interests.

## ETHICS STATEMENT

The ethics application (No. 21KY105) was approved by the Research Ethics Committee of Shijitan Hospital (Beijing, China).

## Supporting information

Figure S1.

Table S1.

## Data Availability

All data analyzed during this study are included in this published article and its Supporting Information files. Supplementary materials (figures, tables, scripts, graphical abstract, slides, videos, Chinese translated versions and update materials) may be found in the online DOI or iMeta Science (http://www.imeta.science/).

## References

[1] Wu, Jiayu , Kai Wang , Xuemei Wang , Yanli Pang , and Changtao Jiang . 2021. “The Role of the Gut Microbiome and its Metabolites in Metabolic Diseases.” Protein & Cell 12: 360–73. 10.1007/s13238-020-00814-7 33346905 PMC8106557

[2] Cullin, Nyssa , Camila Azevedo Antunes , Ravid Straussman , Christoph K. Stein‐Thoeringer , and Eran Elinav . 2021. “Microbiome and Cancer.” Cancer Cell 39: 1317–41. 10.1016/j.ccell.2021.08.006 34506740

[3] Witkowski, Marco , Taylor L. Weeks , and Stanley L. Hazen . 2020. “Gut Microbiota and Cardiovascular Disease.” Circulation Research 127: 553–70. 10.1161/circresaha.120.316242 32762536 PMC7416843

[4] Kolodziejczyk, Aleksandra A. , Danping Zheng , Oren Shibolet , and Eran Elinav . 2019. “The Role of the Microbiome in NAFLD and NASH.” EMBO Molecular Medicine 11: e9302. 10.15252/emmm.201809302 30591521 PMC6365925

[5] Yu, Jun , Qiang Feng , Sunny Hei Wong , Dongya Zhang , Qiao yi Liang , Youwen Qin , Longqing Tang , et al. 2017. “Metagenomic Analysis of Faecal Microbiome as a Tool Towards Targeted Non‐Invasive Biomarkers for Colorectal Cancer.” Gut 66: 70–78. 10.1136/gutjnl-2015-309800 26408641

[6] Lynch, Susan V. , and Oluf Pedersen . 2016. “The Human Intestinal Microbiome in Health and Disease.” New England Journal of Medicine 375: 2369–79. 10.1056/NEJMra1600266 27974040

[7] Hajishengallis, George . 2014. “Immunomicrobial Pathogenesis of Periodontitis: Keystones, Pathobionts, and Host Response.” Trends in Immunology 35: 3–11. 10.1016/j.it.2013.09.001 24269668 PMC3947349

[8] Sepich‐Poore, Gregory D. , Laurence Zitvogel , Ravid Straussman , Jeff Hasty , Jennifer A. Wargo , and Rob Knight . 2021. “The Microbiome and Human Cancer.” Science 371: eabc4552. 10.1126/science.abc4552 33766858 PMC8767999

[9] Zhu, Xinyi , Henghui Li , Liuzhu Zhou , Huijun Jiang , Minghui Ji , and Jin Chen . 2023. “Evaluation of the Gut Microbiome Alterations in Healthy Rats After Dietary Exposure to Different Synthetic ZnO Nanoparticles.” Life Sciences 312: 121250. 10.1016/j.lfs.2022.121250 36455650

[10] Ling, Zongxin , Hang Xiao , and Wei Chen . 2022. “Gut Microbiome: The Cornerstone of Life and Health.” Advanced Gut & Microbiome Research 2022: 9894812. 10.1155/2022/9894812

[11] Portincasa, Piero , Leonilde Bonfrate , Mohamad Khalil , Maria D. Angelis , Francesco M. Calabrese , Mauro D'Amato , David Q. H. Wang , and Agostino Di Ciaula . 2022. “Intestinal Barrier and Permeability in Health, Obesity and NAFLD.” Biomedicines 10: 83. 10.3390/biomedicines10010083 PMC8773010 35052763

[12] Chen, Ju , Elise Pitmon , and Kepeng Wang . 2017. “Microbiome, Inflammation and Colorectal Cancer.” Seminars in Immunology 32: 43–53. 10.1016/j.smim.2017.09.006 28982615

[13] Sochocka, Marta , Katarzyna Donskow‐Łysoniewska , Breno Satler Diniz , Donata Kurpas , Ewa Brzozowska , and Jerzy Leszek . 2019. “The Gut Microbiome Alterations and Inflammation‐Driven Pathogenesis of Alzheimer's Disease—A Critical Review.” Molecular Neurobiology 56: 1841–51. 10.1007/s12035-018-1188-4 29936690 PMC6394610

[14] Martel, Jan , Chang Shih‐Hsin , Yun‐Fei Ko , Tsong‐Long Hwang , John D. Young , and David M. Ojcius . 2022. “Gut Barrier Disruption and Chronic Disease.” Trends in Endocrinology & Metabolism 33: 247–65. 10.1016/j.tem.2022.01.002 35151560

[15] Genua, Flavia , Vedhika Raghunathan , Mazda Jenab , William M. Gallagher , and David J. Hughes . 2021. “The Role of Gut Barrier Dysfunction and Microbiome Dysbiosis in Colorectal Cancer Development.” Frontiers in Oncology 11: 626349. 10.3389/fonc.2021.626349 33937029 PMC8082020

[16] Yu, Linda Chia‐Hui . 2018. “Microbiota Dysbiosis and Barrier Dysfunction in Inflammatory Bowel Disease and Colorectal Cancers: Exploring a Common Ground Hypothesis.” Journal of Biomedical Science 25: 79. 10.1186/s12929-018-0483-8 30413188 PMC6234774

[17] Wang, Zhouli , Rui Cai , Zhenpeng Gao , Yahong Yuan , and Tianli Yue . 2020. “Immunomagnetic Separation: An Effective Pretreatment Technology for Isolation and Enrichment in Food Microorganisms Detection.” Comprehensive Reviews in Food Science and Food Safety 19: 3802–24. 10.1111/1541-4337.12656 33337037

[18] Olsvik, Ørjan , Tanja Popovic , Eystein Skjerve , Kofitsyo S. Cudjoe , Erik Hornes , John Ugelstad , and Mathias Uhlén . 1994. “Magnetic Separation Techniques in Diagnostic Microbiology.” Clinical Microbiology Reviews 7: 43–54. 10.1128/CMR.7.1.43 8118790 PMC358305

[19] Loftus, Mark , Sayf Al‐Deen Hassouneh , and Shibu Yooseph . 2021. “Bacterial Community Structure Alterations Within the Colorectal Cancer Gut Microbiome.” BMC Microbiology 21: 98. 10.1186/s12866-021-02153-x 33789570 PMC8011136

[20] Tilg, Herbert , Timon E. Adolph , Romana R. Gerner , and Alexander R. Moschen . 2018. “The Intestinal Microbiota in Colorectal Cancer.” Cancer Cell 33: 954–64. 10.1016/j.ccell.2018.03.004 29657127

[21] Kurt, Melike , and Zeki Yumuk . 2021. “Diagnostic Accuracy of *Fusobacterium nucleatum* IgA and IgG ELISA Test in Colorectal Cancer.” Science Report 11: 1608. 10.1038/s41598-021-81171-1 PMC7811007 33452405

[22] Chang, Yuxiao , Fengyi Hou , Zhiyuan Pan , Zongyu Huang , Ni Han , Lei Bin , Huimin Deng , et al. 2019. “Optimization of Culturomics Strategy in Human Fecal Samples.” Frontiers in Microbiology 10: 10. 10.3389/fmicb.2019.02891 31921067 PMC6927924

[23] Lopès, Amélie , Elisabeth Billard , Al Hassan Casse , Romain Villéger , Julie Veziant , Gwenaëlle Roche , Guillaume Carrier , et al. 2020. “Colibactin‐Positive *Escherichia coli* Induce a Procarcinogenic Immune Environment Leading to Immunotherapy Resistance in Colorectal Cancer.” International Journal of Cancer 146: 3147–59. 10.1002/ijc.32920 32037530

[24] Long, Xiaohang , Chi Chun Wong , Li Tong , Eagle S. H. Chu , Chun Ho Szeto , Minne Y. Y. Go , Olabisi Oluwabukola Coker , et al. 2019. “ *Peptostreptococcus anaerobius* Promotes Colorectal Carcinogenesis and Modulates Tumour Immunity.” Nature Microbiology 4: 2319–30. 10.1038/s41564-019-0541-3 31501538

[25] Kumar, Ritesh , Jennifer L. Herold , Deborah Schady , Jennifer Davis , Scott Kopetz , Margarita Martinez‐Moczygemba , Barbara E. Murray , et al. 2017. “ *Streptococcus gallolyticus* subsp. *gallolyticus* Promotes Colorectal Tumor Development.” PLoS Pathogens 13: e1006440. 10.1371/journal.ppat.1006440 28704539 PMC5509344

[26] Ma, Yongshun , Yao Zhang , Houxiang Jiang , Shixin Xiang , Yueshui Zhao , Mintao Xiao , and Fukuan Du , et al. 2021. “Metagenome Analysis of Intestinal Bacteria in Healthy People, Patients With Inflammatory Bowel Disease and Colorectal Cancer.” Frontiers in Cellular and Infection Microbiology 11: 599734. 10.3389/fcimb.2021.599734 33738265 PMC7962608

[27] Flemer, Burkhardt , Denise B. Lynch , Jillian M. R. Brown , Ian B. Jeffery , Feargal J. Ryan , Marcus J. Claesson , Micheal O'Riordain , Fergus Shanahan , and Paul W. O'Toole . 2017. “Tumour‐Associated and Non‐Tumour‐Associated Microbiota in Colorectal Cancer.” Gut 66: 633–43. 10.1136/gutjnl-2015-309595 26992426 PMC5529966

[28] Armstrong, Heather , Misagh Alipour , Rosica Valcheva , Michael Bording‐Jorgensen , Juan Jovel , Deenaz Zaidi , Prachi Shah , et al. 2019. “Host Immunoglobulin G Selectively Identifies Pathobionts in Pediatric Inflammatory Bowel Diseases.” Microbiome 7: 1. 10.1186/s40168-018-0604-3 30606251 PMC6317230

[29] Lagier, Jean‐Christophe , Grégory Dubourg , Matthieu Million , Frédéric Cadoret , Melhem Bilen , Florence Fenollar , Anthony Levasseur , et al. 2018. “Culturing the Human Microbiota and Culturomics.” Nature Reviews Microbiology 16: 540–50. 10.1038/s41579-018-0041-0 29937540

[30] Saus, Ester , Susana Iraola‐Guzmán , Jesse R. Willis , Anna Brunet‐Vega , and Toni Gabaldón . 2019. “Microbiome and Colorectal Cancer: Roles in Carcinogenesis and Clinical Potential.” Molecular Aspects of Medicine 69: 93–106. 10.1016/j.mam.2019.05.001 31082399 PMC6856719

[31] Cross, Karissa L. , James H. Campbell , Manasi Balachandran , Alisha G. Campbell , Connor J. Cooper , Ann Griffen , Matthew Heaton , et al. 2019. “Targeted Isolation and Cultivation of Uncultivated Bacteria by Reverse Genomics.” Nature Biotechnology 37: 1314–21. 10.1038/s41587-019-0260-6 PMC6858544 31570900

[32] Heidari Baladehi, Mohammadhadi , Maryam Hekmatara , Yuehui He , Yogendra Bhaskar , Zengbin Wang , Lu Liu , Yuetong Ji , and Jian Xu . 2021. “Culture‐Free Identification and Metabolic Profiling of Microalgal Single Cells via Ensemble Learning of Ramanomes.” Analytical Chemistry 93: 8872–80. 10.1021/acs.analchem.1c01015 34142549

[33] Bu, Shengjun , Kuiyu Wang , Chengyu Wang , Zhongyi Li , Zhuo Hao , Wensen Liu , and Jiayu Wan . 2020. “Immunoassay for Foodborne Pathogenic Bacteria Using Magnetic Composites Ab@Fe_3_O_4_, Signal Composites Ap@PtNp, and Thermometer Readings.” Microchimica Acta 187: 679. 10.1007/s00604-020-04657-1 33247373

[34] Ogizbayeva, Alina , and Yermek Turgunov . 2021. “Bacterial Translocation in Colorectal Cancer Patients.” Journal of Clinical Medicine of Kazakhstan 18: 8–13. 10.23950/jcmk/10926

[35] Kwong, Thomas N. Y. , Xiansong Wang , Geicho Nakatsu , Tai Cheong Chow , Timothy Tipoe , Rudin Z. W. Dai , Kelvin K. K. Tsoi , et al. 2018. “Association Between Bacteremia from Specific Microbes and Subsequent Diagnosis of Colorectal Cancer.” Gastroenterology 155: 383–90.e8. 10.1053/j.gastro.2018.04.028 29729257

[36] Velmurugan, Ganesan , Vasudevan Dinakaran , Jeyaprakash Rajendhran , and Krishnan Swaminathan . 2020. “Blood Microbiota and Circulating Microbial Metabolites in Diabetes and Cardiovascular Disease.” Trends in Endocrinology & Metabolism 31: 835–47. 10.1016/j.tem.2020.01.013 33086076

[37] Mutignani, Massimiliano , Roberto Penagini , Giorgio Gargari , Simone Guglielmetti , Marcello Cintolo , Aldo Airoldi , and Pierfrancesco Leone , et al. 2021. “Blood Bacterial DNA, Intestinal Adenoma and Colorectal Cancer.” Cancers 13: 6363. 10.1101/2021.07.22.21260498 34944982

[38] Hand, Timothy W. , and Andrea Reboldi . 2021. “Production and Function of Immunoglobulin A.” Annual Review of Immunology 39: 695–718. 10.1146/annurev-immunol-102119-074236 33646857

[39] Chakaroun, Rima M. , Lucas Massier , Anna Heintz‐Buschart , Nedal Said , Joerg Fallmann , Alyce Crane , Tatjana Schütz , et al. 2021. “Circulating Bacterial Signature is Linked to Metabolic Disease and Shifts With Metabolic Alleviation After Bariatric Surgery.” Genome Medicine 13: 105. 10.1186/s13073-021-00919-6 34158092 PMC8218394

[40] Dinakaran, Vasudevan , Andiappan Rathinavel , Muthuirulan Pushpanathan , Ramamoorthy Sivakumar , Paramasamy Gunasekaran , and Jeyaprakash Rajendhran . 2014. “Elevated Levels of Circulating DNA in Cardiovascular Disease Patients: Metagenomic Profiling of Microbiome in the Circulation.” PLoS One 9: e105221. 10.1371/journal.pone.0105221 25133738 PMC4136842

[41] Schierwagen, Robert , Camila Alvarez‐Silva , Mette Simone Aae Madsen , Carl Christian Kolbe , Carsten Meyer , Daniel Thomas , Frank Erhard Uschner , et al. 2019. “Circulating Microbiome in Blood of Different Circulatory Compartments.” Gut 68: 578–80. 10.1136/gutjnl-2018-316227 29581241

[42] Guilhot, Elodie , Saber Khelaifia , Bernard La Scola , Didier Raoult , and Grégory Dubourg . 2018. “Methods for Culturing Anaerobes from Human Specimen.” Future Microbiology 13: 369–81. 10.2217/fmb-2017-0170 29446650

[43] Lagier, Jean‐Christophe , Sophie Edouard , Isabelle Pagnier , Oleg Mediannikov , Michel Drancourt , and Didier Raoult . 2015. “Current and Past Strategies for Bacterial Culture in Clinical Microbiology.” Clinical Microbiology Reviews 28: 208–36. 10.1128/CMR.00110-14 25567228 PMC4284306

[44] Tidjani Alou, Maryam , Sabrina Naud , Saber Khelaifia , Marion Bonnet , Jean‐Christophe Lagier , and Didier Raoult . 2020. “State of the Art in the Culture of the Human Microbiota: New Interests and Strategies.” Clinical Microbiology Reviews 34: e00129–19. 10.1128/CMR.00129-19 33115723 PMC7605308

[45] Browne, Hilary P. , Samuel C. Forster , Blessing O. Anonye , Nitin Kumar , B. Anne Neville , Mark D. Stares , David Goulding , and Trevor D. Lawley . 2016. “Culturing of ‘Unculturable’ Human Microbiota Reveals Novel Taxa and Extensive Sporulation.” Nature 533: 543–6. 10.1038/nature17645 27144353 PMC4890681

[46] Abdulhussien, Zainab . 2018. “Antimicrobial Effect of Pyocyanin Extracted from *Pseudomonas aeroginosa* (2).” European Journal of Experimental Biology 6: 1–4.

