# Supplementary material for: Immunomagnetic‐bead enriched culturomics (IMBEC) for isolating pathobionts from feces of colorectal cancer patients
Source: Imeta. 2023 Apr 5;2(2):e100. doi: 10.1002/imt2.100 (PMC10989793; doi:10.1002/imt2.100)
Supplement: Supplementary file 1 — Figure S1. [file IMT2-2-e100-s002.docx]

# Immunomagnetic-bead enriched culturomics (IMBEC) for isolating pathobionts from feces of colorectal cancer patients

**Running title: IMBEC for isolating pathobionts from CRC patients**

Ziran Huang^1,2,#^, Yuxiao Chang^2,#^, Kun Hao^3,#^, Yafang Tan^2,3^, Lei Ding^4^, Likun Wang^2^, Zhen Wang^2^, Zhiyuan Pan^2^, Hong Gao^4^, Jiahong Wu^1, *^, Yubing Zhu^4, *^, Qi Gao^3,*^, Yujing Bi^2,3,*^, Ruifu Yang^2,3^

^1^ The Key and Characteristic Laboratory of Modern Pathogen Biology, School of Basic Medical Sciences, Guizhou Medical University, Guiyang, China

^2^ State Key Laboratory of Pathogen and Biosecurity, Beijing Institute of Microbiology and Epidemiology, Beijing 100071, China

^3^ Beijing Key Laboratory of POCT for Bioemergency and Clinic (BZ0329), Beijing 100071, China;

^4^ Beijing Shijitan Hospital, Capital Medical University, Beijing 100038, China

#These authors contributed equally to this work.

* Corresponding authors: Jiahong Wu (jiahongw@gmc.edu.cn)

Qi Gao (qi.gao@hotgen.com.cn)

Yubing Zhu [(282737385@qq.com)](mailto:(282737385@qq.com))

Yujing Bi (byj7801@sina.com)

**
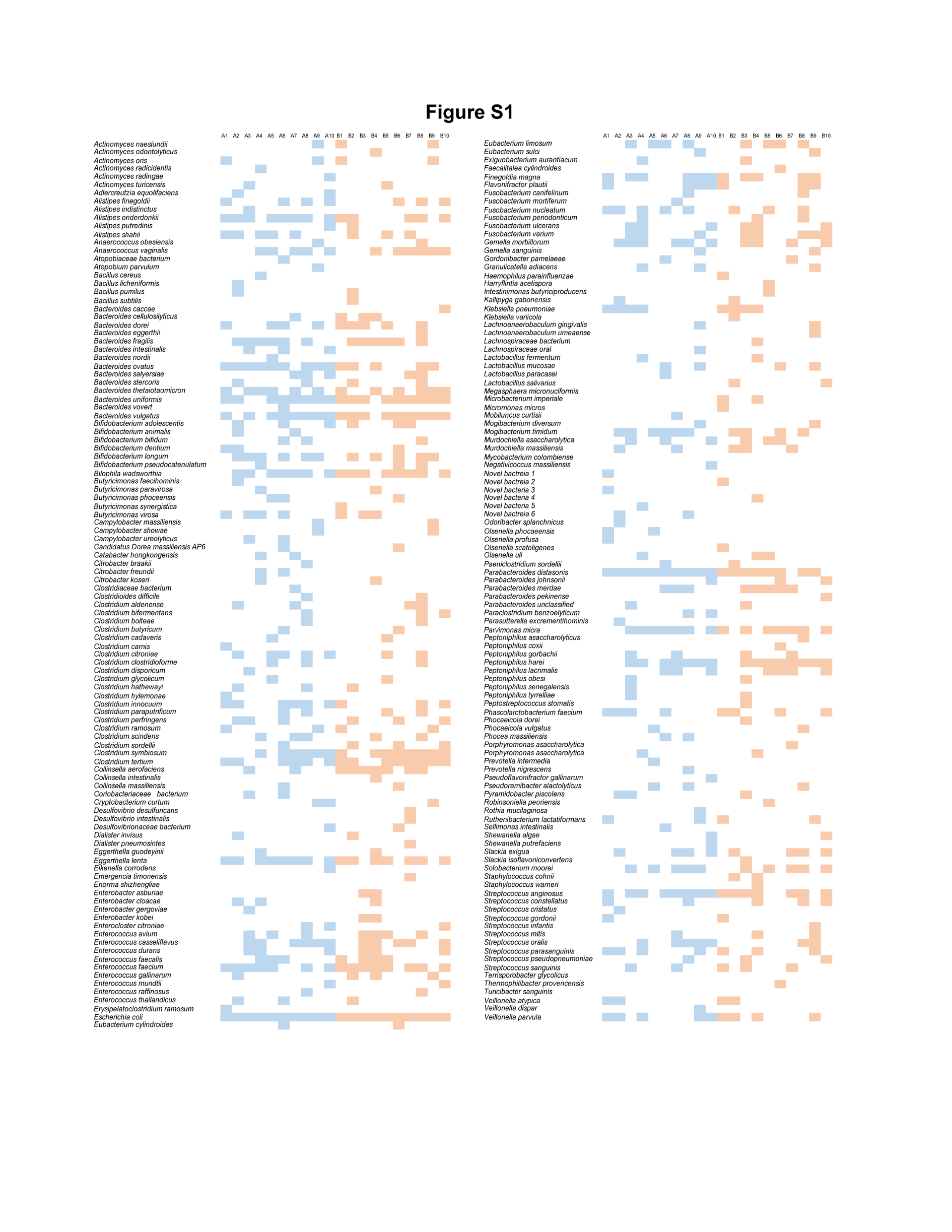
**

**Figure S1** Distribution of 209 species in 10 samples. The blue color represents the bacteria isolated in the antibody group and the orange color in the blank one.


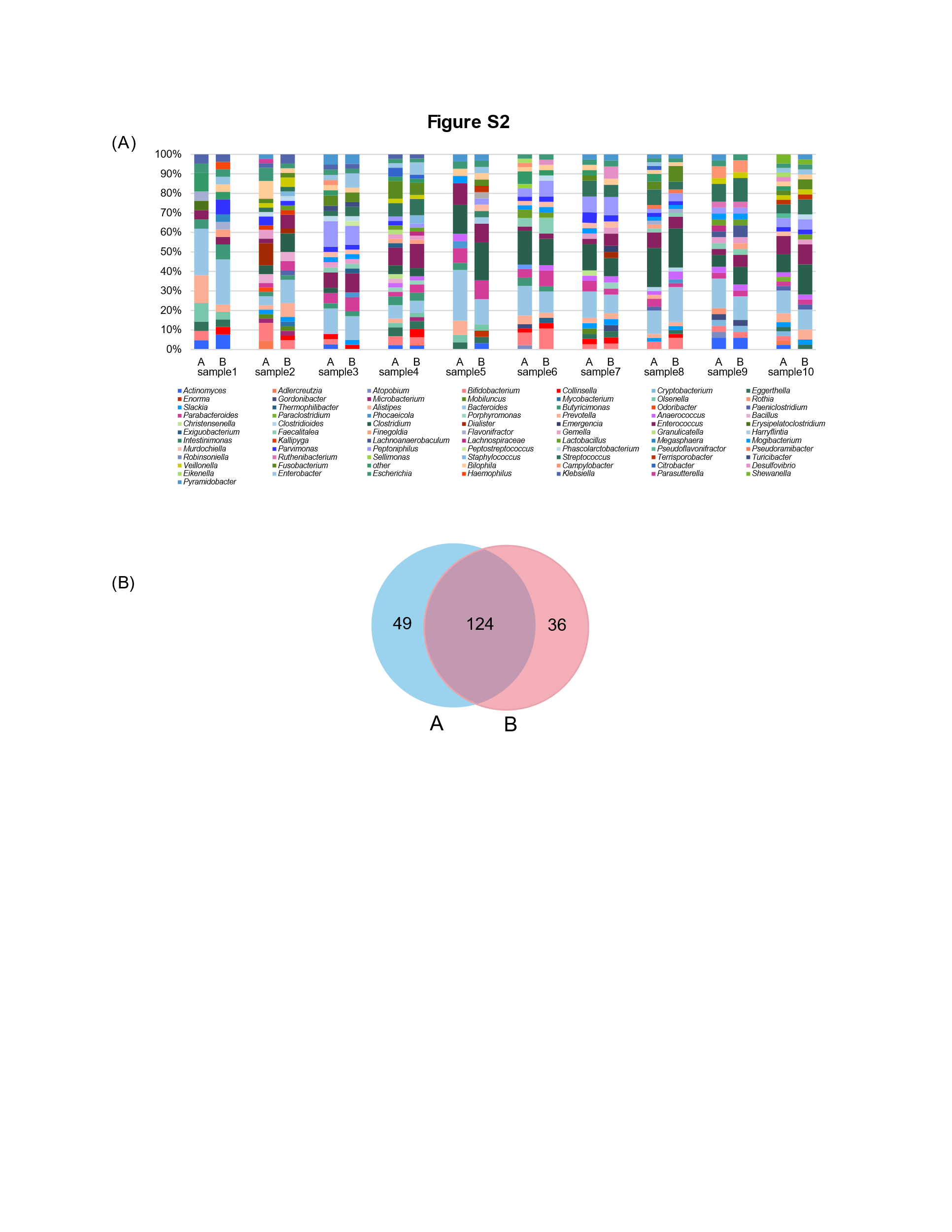


**Figure S2** Composition and number of species isolated in the antibody or blank group. **(**A) Bacterial composition at genus level of A and B in each sample. (B) Number of bacterial species isolated from A and B. A: antibody group, B: blank group.


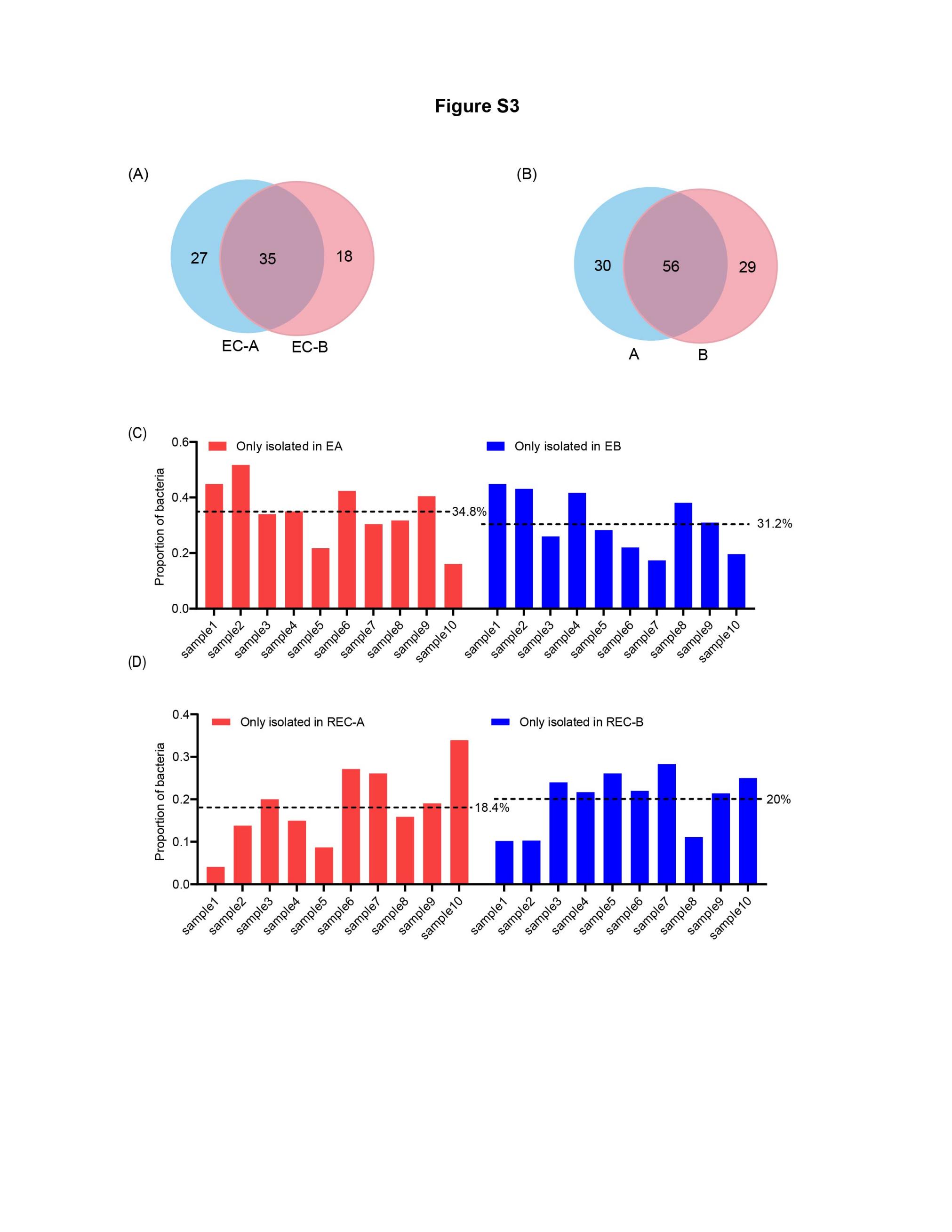


**Figure S3** Comparison of species isolated from the antibody or blank group under different condition. (A)-(B) Number of bacterial species isolated from A or B under different condition. (A) EC culture condition. (B) EC and REC culture condition. (C)-(D) Proportion of bacteria only isolated in A or B under EC or REC conditions. (C) EC culture condition. (D) REC culture condition. Dashed lines represent the mean values. A: antibody group (EC-A+REC-A); B: blank group (EC-B+REC-B). EC-A: enrichment culture of antibody group; EC-B: enrichment culture of blank group; REC-A: re-enrichment culture of antibody group; REC-B: re-enrichment culture of blank group.


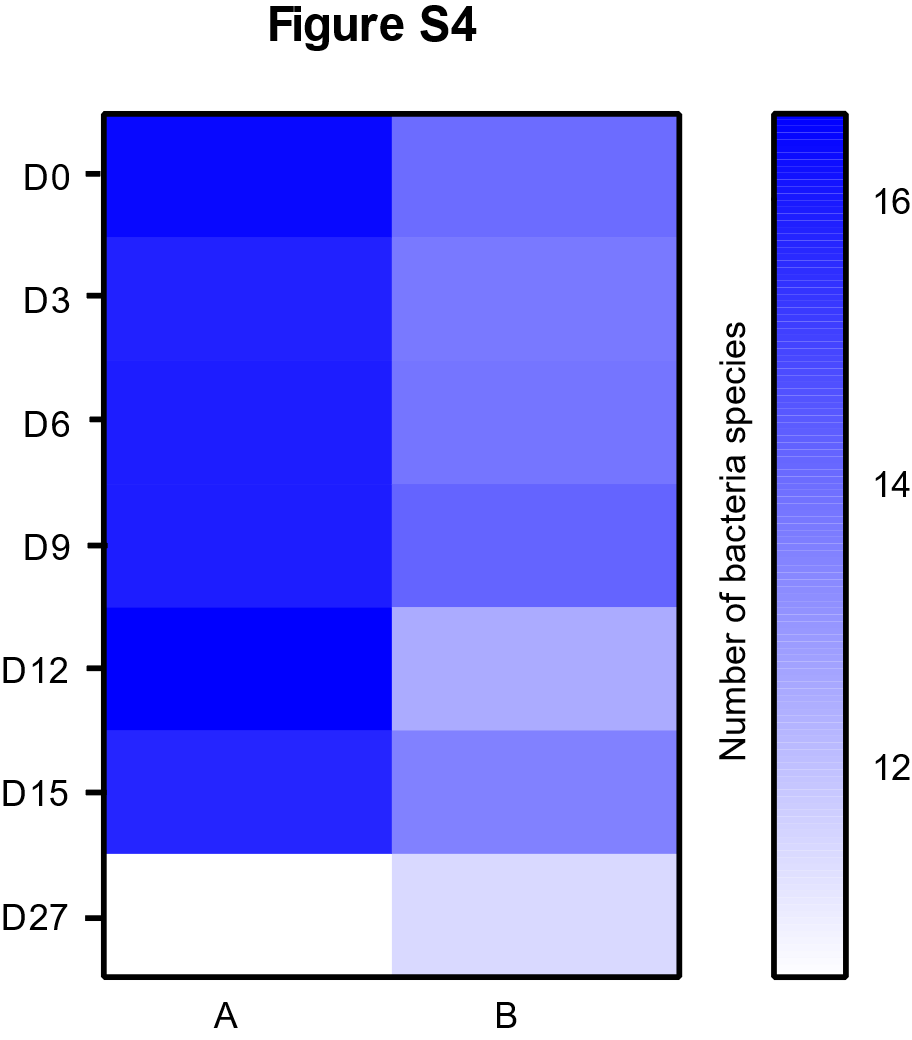


**Figure S4** Heat map of the number of bacteria isolated at each time point in the antibody group or the blank group in 10 samples. A: antibody group, B: blank group.


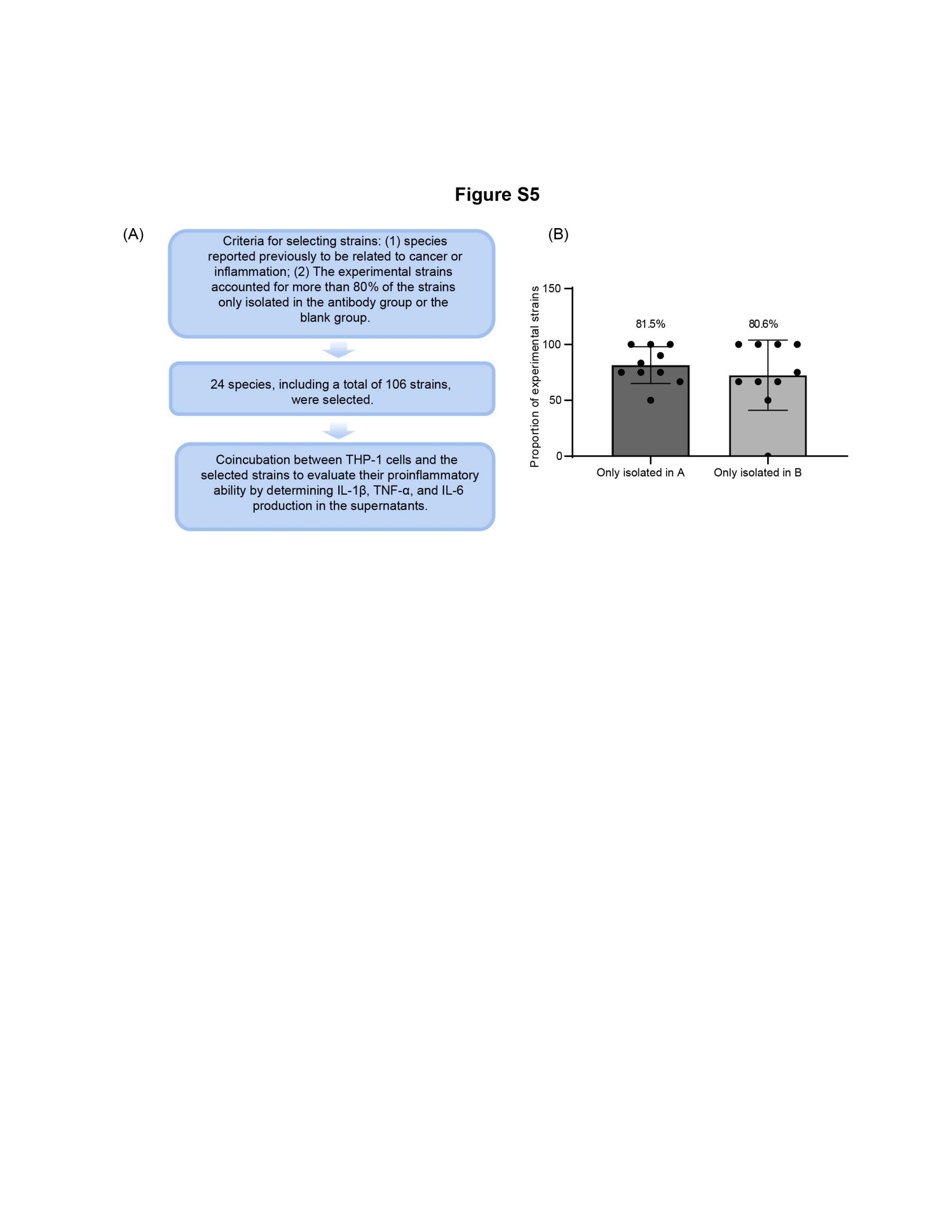


**Figure S5** The criteria were used to select experimental strains from the antibody and blank group. (A) Flow chart of selecting bacterial strains for co-incubation with THP-1 cells. (B) Percentage of strains per sample selected from both groups. Data are presented as mean ± SD, two-tailed unpaired Student’s t-test. A: antibody group, B: blank group.


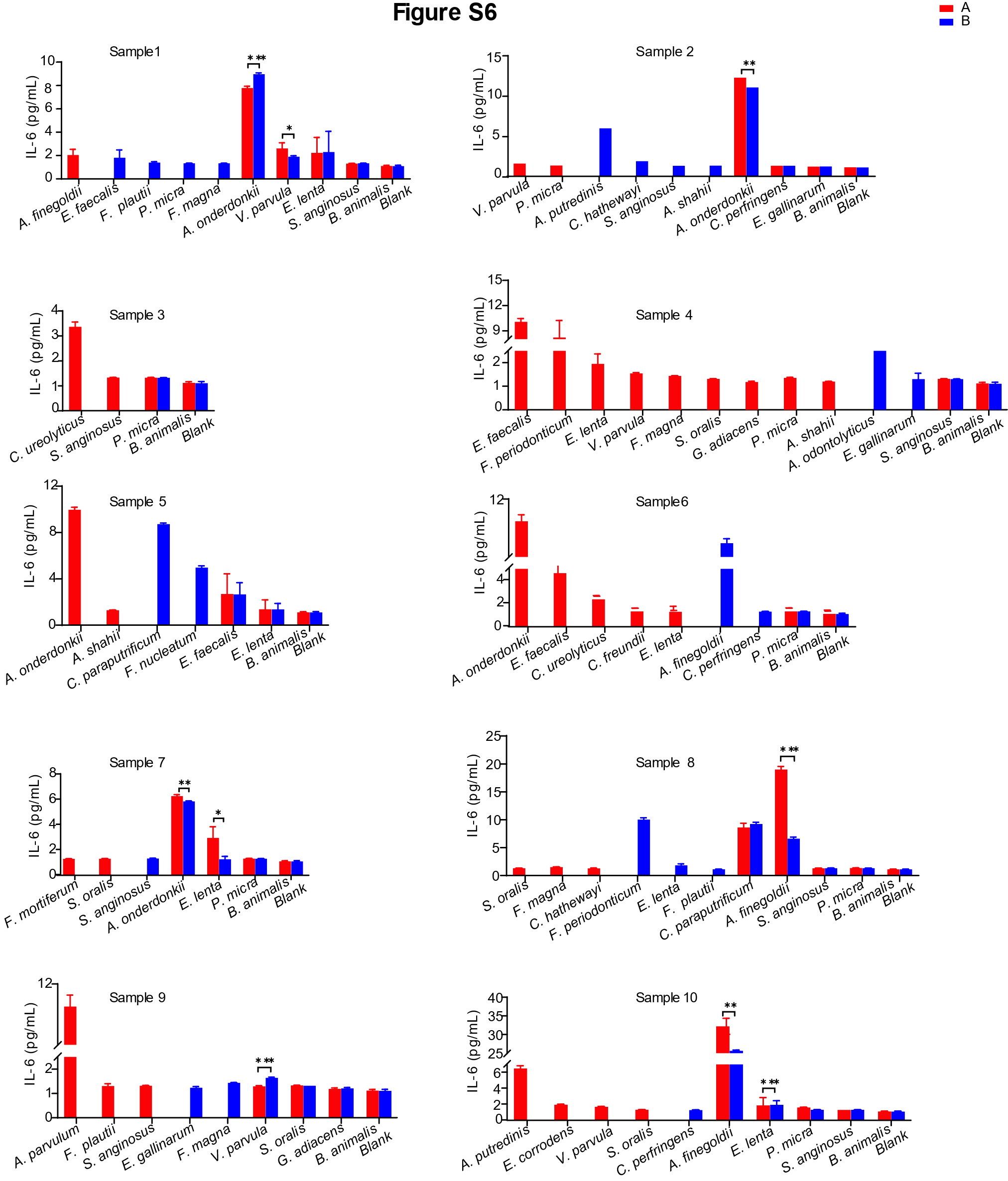


**Figure S6** IL-6 content in the supernatant of 106 strains co-incubated with THP-1. Data are presented as mean ± SD, two-tailed unpaired Student’s t-test, * *p*<0.05, ** *p*<0.01,*** *p*<0.001. A: antibody group, B: blank group.


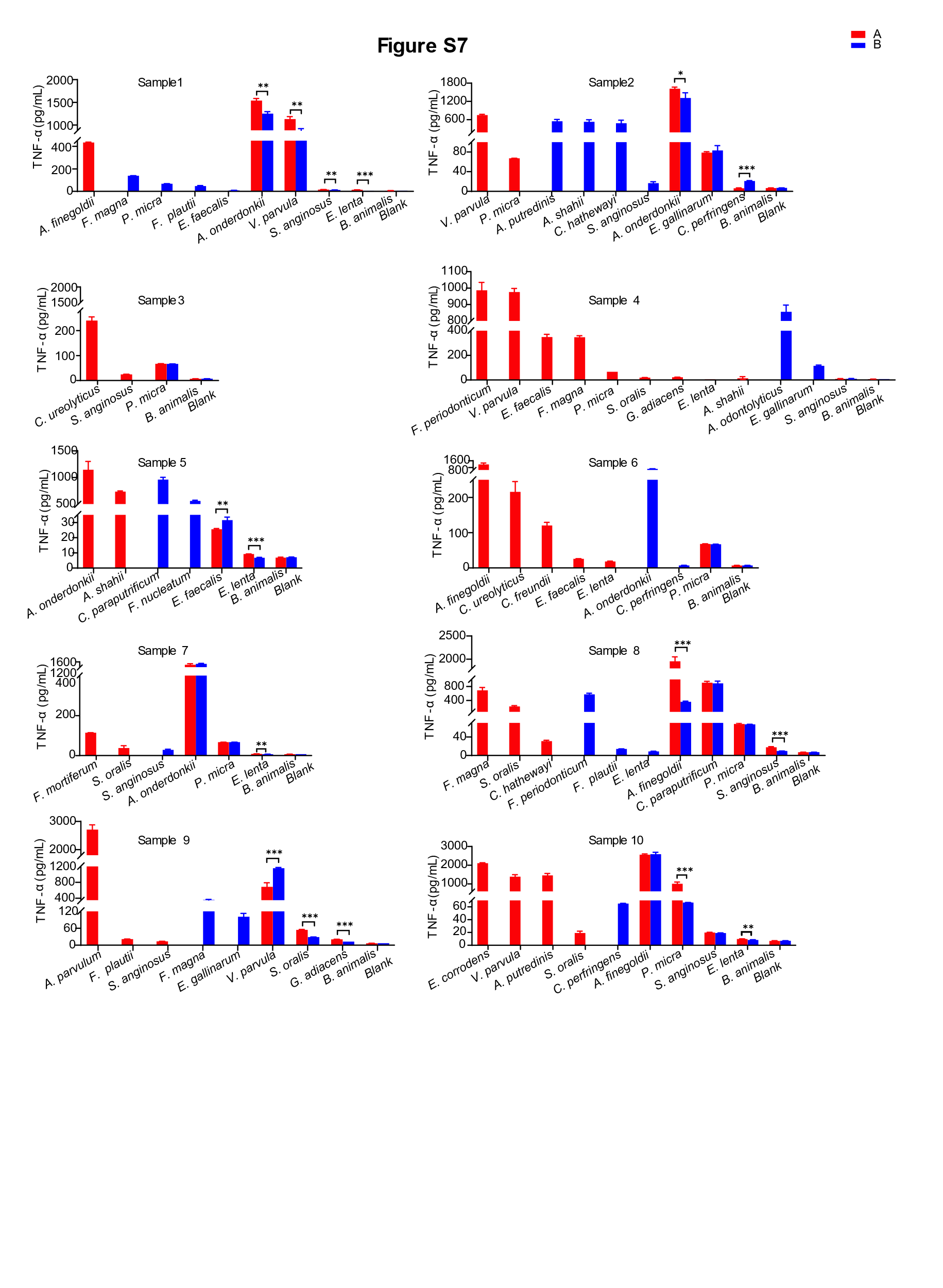


**Figure S7** TNF-α content in the supernatant of 106 strains co-incubated with THP-1. Data are presented as mean ±SD, two-tailed unpaired Student’s t-test, * *p*<0.05, ** *p*<0.01,*** *p*<0.001. A: antibody group, B: blank group.

**
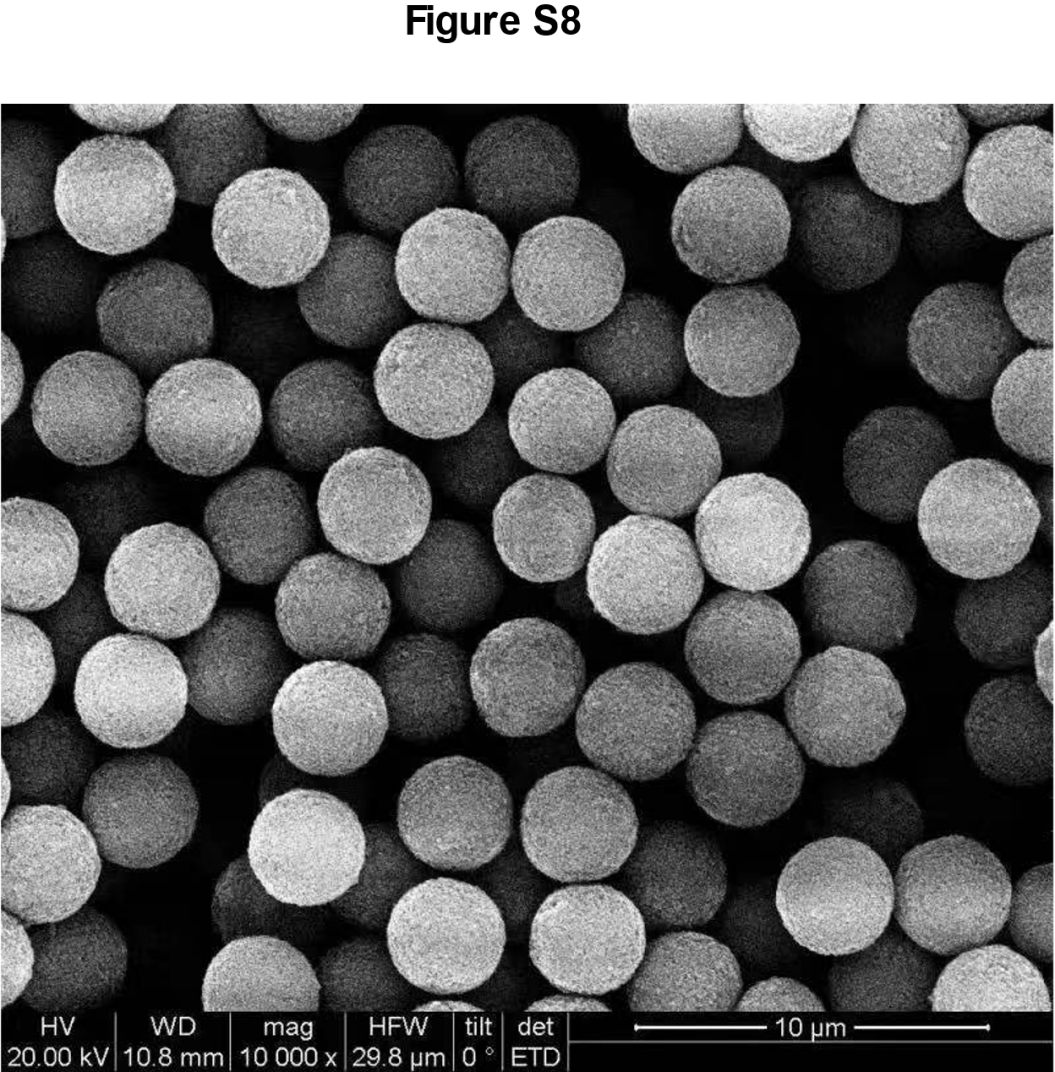
**

**Figure S8** TEM was used to evaluate the morphological features of the magnetic beads.
